# Mesoporous Membrane Materials Based on Ultra-High-Molecular-Weight Polyethylene: From Synthesis to Applied Aspects

**DOI:** 10.3390/membranes11110834

**Published:** 2021-10-28

**Authors:** Olga V. Arzhakova, Andrei I. Nazarov, Arina R. Solovei, Alla A. Dolgova, Aleksandr Yu. Kopnov, Denis K. Chaplygin, Polina M. Tyubaeva, Alena Yu. Yarysheva

**Affiliations:** 1 Faculty of Chtmistry, Lomonosov Moscow State University, Leninskie Gory 1/3, 119991 Moscow, Russia; andrey-nazarov96@yandex.ru (A.I.N.); arina.solovey.2002@mail.ru (A.R.S.); dolgova2003@mail.ru (A.A.D.); kopnov2000@yandex.ru (A.Y.K.); denkryto@mail.ru (D.K.C.); alyonusha@gmail.com (A.Y.Y.); 2 Chemistry of Innovative Materials and Technologies, Plekhanov Russian University of Economics, Stremyanny Lane 36, 117997 Moscow, Russia; polina-tyubaeva@yandex.ru

**Keywords:** mesoporous polymer materials, ultra-high-molecular-weight polyethylene, environmental crazing, water vapor permeability, breathable materials, polymeric membranes

## Abstract

The development of new porous polymeric materials with nanoscale pore dimensions and controlled morphology presents a challenging problem of modern materials and membrane science, which should be based on scientifically justified approaches with the emphasis on ecological issues. This work offers a facile and sustainable strategy allowing preparation of porous nanostructured materials based on ultra-high-molecular-weight polyethylene (UHMWPE) via the mechanism of environmental intercrystallite crazing and their detailed characterization by diverse physicochemical methods, including SEM, TEM, AFM, liquid and gas permeability, DSC, etc. The resultant porous UHMWPE materials are characterized by high porosity (up to ~45%), pore interconnectivity, nanoscale pore dimensions (below 10 nm), high water vapor permeability [1700 g/(m^2^ × day)] and high gas permeability (the Gurley number ~300 s), selectivity, and good mechanical properties. The applied benefits of the advanced UHMWPE mesoporous materials as efficient membranes, breathable, waterproof, and insulating materials, light-weight materials with reduced density, gas capture and storage systems, porous substrates and scaffolds are discussed.

## 1. Introduction

Scientific challenges of the materials science of XXI^th^ century are directed towards controlled design and tailoring of innovative high-performance polymeric materials with desired properties and task-specific applications, including the preparation of porous organic materials that are in great demand [1,2,3,4,5,6,7] in diverse areas of science, technology, and even in our daily life. Nowadays, porous organic materials prove their evident applied value as they can be used as filtration/separation membrane materials for ultra/nano/microfiltration, membrane distillation, hemodialysis, and environmental remediation, breathable materials, sorbents, insulating and light-weight materials, high-performance filters, fuel cell membranes and battery separators, gas storage and separation materials, materials for capture and storage of clean fuels such as hydrogen (H_2_) and methane (CH_4_), packaging materials [3,7,8,9,10] as well as matrixes for the accommodation of encapsulation agents and preparation of the innovative materials with controlled drug delivery and release, systems for catalysis, sensors, precursors for nanostructured carbon materials, supports for biomolecular immobilization and cell scaffolds [4,5,11].

Depending on their pore size, porous materials are categorized into three classes by the IUPAC classification: macroporous (pore size >50 nm), mesoporous (pore size = 2–50 nm) and microporous (pore size < 2 nm) materials [12]. In general practice, porous organic materials are fabricated by using different modern technologies that combine the resources of polymer chemistry and physics. These methods include interface polymerization, suspension polymerization, emulsion polymerization, polycondensation, hyper-crosslinking, templating, thermally induced phase separation (TIPS), phase inversion, air casting of polymer solutions, precipitation of vapor phase, etching, immersion precipitation, dry, wet, stretching, and electrospinning [4,6,7]. In common practice, each particular methodhas its own potential with intrinsic benefits and limitations that are rooted in the nature of starting polymers, optimal technological solutions with respect to ecological safety, economic issues, and task-specific applications. Nowadays, as was highlighted in the recent review, “…the field of porous polymers is at an exciting stage of its evolution” [7]. The main requirements to high-performance and innovative porous materials can be briefly summarized as follows: uniform porosity, high surface area, scalable and uniform pore dimensions, pore interconnectivity, chemical and thermal stability, good mechanical characteristics, etc. In this connection, semicrystalline polymers with their evident benefits due to the long-range order are in the spotlight and embraced the attention of many scientists.

Among diverse commercial semicrystalline polymers with their impressive list of achievements, UHMWPE is distinguished due to its unique physicochemical characteristics and related applied bonuses. Generally speaking, UHMWPE is a member of well-respected and trusted polyethylene family with the repeat unit [C_2_H_4_]_n_, with *n* denoting the degree of polymerization (for UHMWPE, the minimal degree of polymerization is n ≈ 36,000) [13]. UHMWPE is a semicrystalline polymer containing crystalline and amorphous phases when crystalline phase is presented by well-organized crystalline lamellae with folded chains as first recognized by Keller [14,15]. Amorphous phase is accommodated in the space between crystalline lamellae and contains load-bearing tie molecules that link the neighboring crystallites.

Among the abundant diversity of polymers with their task-oriented properties, UHMWPE occupies its own unique niche due to its outstanding characteristics, including biocompatibility, high wear-resistance and tribological performance, ductility, chemical stability, abrasion resistance, superior impact resistance, bioinertness, biomedical properties, excellent mechanical properties, etc. [13,16,17,18].

Of special interest are porous materials based on UHMWPE, which offer substantial advantages for their practical use in different applications, including membrane technology, engineering, medicine and biotechnology, water cleaning, oil spill recovery, gas capture and storage, elimination of water pollutions, tissue engineering and control over proliferation and differentiation in living cell populations [5,19,20,21,22].

However, as compared with traditional polymers, preparation of porous UHMWPE-based materials by conventional methods is naturally aggravated by multiple complications due to its exceptionally high molecular weight, high melt viscosity and poor processability. At the present time, diverse techniques have been developed for the preparation of UHMWPE-based porous materials such as powder sintering method, thermally induced phase separation (TIPS) method, non-dense injection molding (NIM) method, porogen method, salt leaching [22,23,24,25,26,27]. However, the TIPS process involves high consumption of toxic solvents and extractants, thus creating serious ecological complications. Porous UHMWPE materials can be also fabricated by so-called porogen method using diverse porogens (including salts, waxes) and extractants [22,28,29], but the potential of this approach is critically limited by the multistage scenario, strict temperature control, and high consumption of ecologically unfriendly reagents. The method of powder sintering also allows preparation of efficient UHMWPE porous membranes [24] but this approach involves several stages, long molding cycle, and low production efficiency.

Therefore, the search for new sustainable and ecologically friendly methods for the preparation of porous (mesoporous) UHMWPE materials presents a challenging multidisciplinary task. In this connection, environmental crazing of solid polymers can be envisaged as an alternativeand efficient approach allowing preparation of porous UHMWPE materials. This strategy is based on the fundamental property of solid polymers to experience stress-induced structural self-organization via cavitation and fibrillation due to deformation in the presence of physically active liquid environments (PALE) [30,31,32]. In addition to the academic background, environmental crazing offers a straight and powerful strategy for controlled design of “difficult” UHMWPE and preparation of nanostructured porous materials with high porosity and good mechanical characteristics, which can serve as sorbents, membranes, breathable materials, scaffolds and supports for the development of diverse multifunctional nanocomposite polymeric materials containing target additives incorporated within mesoporous cavities [30,31,32,33,34].

This work offers the sustainable and ecologically safe route for the development of a new family of porous UHMWPE materials via environmental crazing and highlights the benefits and applied value of this approach.

## 2. Materials and Methods

### 2.1. Materials

The films of UHMWPE (DSM, Sittard, The Netherlands) were used; the film thickness was 200 µm; *M_n_*~10^6^. As physically active liquid environments (PALE), we used *n*-heptane, *n*-decane, and isopropanol (IPA) (Sigma-Aldrich, St. Louis, MI, USA).

As contrasting colorants, Rhodamine 6G (RH6G) and Sudan IV (Sigma-Aldrich, St. Louis, MI, USA) were used.

### 2.2. Preparation of Biphase Oil-in-Water Emulsions

Lyophobic oil-in-water emulsions with high water content were prepared according to the following procedure: the prescribed amounts of water and the PALE (*n*-heptane, *n*-decane) were placed into a glass vessel (95 parts of water and 5 parts of oil, by volume; 95 vol. % O/W) and the biphase system with a well-defined interfacial boundary between oil and water was subjected to stirring using either a laboratory stirrer or a vortex mixer until the mixture became opaque; the duration of stirring was varied from 10 s to 3 min. The time required for the O/W emulsion to fully recover a single interface boundary between the two phases (oil and water) was ~35 min (breakage of the emulsion). Hence, this survival period is long enough to perform all experiments. After the emulsion breakage, the oil (hydrocarbons) can be easily separated from water. The size of oil droplets was estimated using an Opton microscope (Carl Zeiss AG. Oberkochen, Germany). For better visualization, oil phase (a dispersed phase) was colored with oil-soluble colorants (Sudan IV) whereas water (a continuous phase) was colored by water-soluble colorants (RH6G). The estimated size of spherical oil droplets was 2–4 microns.

### 2.3. Preparation of the Test Samples

The samples with a gage size of 50 mm × 30 mm were cut from the batch roll and stretched at room temperature to a given tensile strain *ε*. The samples were stretched with a constant rate of 5 mm/min to tensile strains *ε* varying from 10 to 400% in the presence of the PALE or the O/W emulsions as the PALE. Tensile strain *ε* was defined as:

(1) $$\varepsilon =\frac{\Delta l}{l_{0}}\times 100\%$$

where Δ*l* is the difference between the final and initial length of the sample, *l*_0_ is the initial length of the sample.

### 2.4. Methods

#### 2.4.1. Differential Scanning Calorimetry (DSC)

The DSC scans were performed on an STA 449 F3 Jupiter thermal analyzer (Netzsch, Selb, Germany). The weight of the test samples was 15 mg. Scanning rate was 20 K/min. The degree of crystallinity *æ_c_* of the semicrystalline samples was estimated according to the standard procedure with the account for the heat of fusion of an ideal crystal (100% crystalline polymer):

*æ*_c_ = ΔH_m_/ΔH^0^_m_ × 100% (2)

where the ΔH_m_ is the heat of fusion of UHMWPE, and ΔH^0^_m_ is the heat of fusion of 100% crystalline UHMWPE, which is equal to 289 J/g [25].

#### 2.4.2. Wide-Angle X-ray Scattering (WAXS)

The UHMWPE samples were studied on a DRON-3M diffractometer (LNPO Burevestnik, St. Petersburg, Russian Federation) (Cu; 20 mA 30 kV; wavelength was 1.541874 Å)

#### 2.4.3. Mechanical Tests

The mechanical tests of the UHMWPE films were performed on a HOUNSFIELD HIKS tensile machine (Hounsfield Test Equipment Ltd., Redhill, UK) at room temperature. The gage size of the samples was 10 mm × 25 mm. Crosshead speed was 5 mm/min. The tests were performed for, at least, 5–7 samples, and the results were averaged. The experimental error did not exceed 3–5%.

#### 2.4.4. Porosity

In the course of tensile drawing of the UHMWPE films, changes in their geometric dimensions (length, thickness, and width) and volume were measured. The accuracy of measurements did not exceed 3–5%. Porosity *W* reads as:

(3) $$W=\frac{\Delta V}{V_{0}+\Delta V}\times 100\%$$

where *V*_0_ is the initial volume of the sample, Δ*V* is the difference between the volume of the deformed sample and the initial sample *V*_0_. The experimental error was 3%.

#### 2.4.5. Liquid Permeability of the Porous UHMWPE Films

To gain a deeper insight into the fine structure of the porous UHMWPE films after their tensile drawing, the method of pressure-driven liquid permeability (PDLP) was applied. Being compared to the classical methods used for the characterization of the structural parameters of porous materials (AFM, scanning electron microscopy, WAXS, SAXS, etc.), the benefits of the PDLP method are primarily related to the fact that so-called “native” structure of the porous samples can be captured when “wet” samples immediately after their stretching in the PALE were tested and the time period between the tensile drawing and the PDLP measurements can be minimized to a few minutes.

After their tensile drawing by a given tensile strain *ε*, the samples under isometric conditions were fastened by contour, thus preventing their shrinkage and side contraction; then, the as-stretched samples were allowed to stay in IPA for 20–25 min. Then, the test samples under isometric conditions were placed into an FMO-2 membrane cell (FMO, Moscow, Russian Federation) filled with IPA. The pressure gradient was 1 bar. The IPA flow rate *Q* was measured for, at least, 3–5 samples. The experimental error in the estimation of the IPA flow did not exceed 3–5%.

Average pore diameter *D_p_* (or pore radius *r*) was calculated according to the Hagen–Poiseuille model when the porous structure is modeled as a network of open channels. Then, the flow rate *Q* reads as

(4) $$Q=\frac{\pi nr^{4}S\Delta P}{8\eta d}=\frac{Wr^{2}S\Delta P}{8\eta d}\;$$

where *n* is the number of pores per unit surface, *r* is the pore radius, Δ*P* is the pressure gradient, *d* is the thickness of the film, *η* is the viscosity of the liquid, and *S* is the membrane area.

Porosity *W* is defined as

(5) $$W=\pi nr^{2}$$

where *n* is the number of pores per unit surface, *r* is the pore radius.

Pore diameter is calculated from Equations (4) and (5) as

(6) $$D_{p}=2\sqrt{\frac{Q8\eta d}{WS\Delta P}}$$

#### 2.4.6. Dye Penetration Tests

Inspection of dye penetration in solids is known to be a reliable non-destructive method of defectoscopy which allows visualization and detection of the pre-existing or stress-induced defects such as pores, cavities, etc. As a contrasting colorant, fluorescent dyes Rhodamine 6G and Sudan IV were used. The UHMWPE films were stretched in the dye-containing solutions (IPA for RH6G and *n*-heptane for Sudan IV) (forced impregnation) or the films after stretching were allowed to stay in the above dye-containing solutions for 30 min at room temperature (passive wet impregnation). Then, for correct visualization, the samples were repeatedly washed with water and ethanol.

#### 2.4.7. Scanning Electron Microscopy (SEM)

Structure of the UHMWPE samples was studied using an EVO 40 XPV scanning electron microscope (Carl Zeiss AG, Oberkochen, Germany). Prior to the SEM observations, the samples were fractured along the stretching direction in liquid nitrogen and decorated with a conducting platinum layer with a thickness of 50–70 nm using a Giko IB-3 setup.

#### 2.4.8. Atomic Force Microscopy (AFM)

Surface morphology of the test samples was studied by the atomic force microscopy on a Multimode microscope with a Nanoscope V controller (Veeco, Plainview, New York, NY, USA). The atomic force microscopy (AFM) observations were performed in air at room temperature under the non-resonance scanning mode (PeakForce Tapping QNM). As probes, the cantilevers based on silicon nitride with a single-crystalline silicon tip SNL-10 (Bruker, Billerica, MA, USA) were used; the nominal resonance frequency was 65 kHz, and the force constant was 0.35 N m^−1^.

#### 2.4.9. Permporometry

Most of the routine characterization methods cannot discriminate between the “active” pores and “inactive” pores present in the skin or in the supporting layer of the membranes. Permporometry offers a reliable characterization method for the evaluation of active pores (below 25 nm) in porous materials [35]. This method is based on the controlled stepwise blocking of pores by vapor condensation and the simultaneous measurement of the gas flux (oxygen permeance) through the membrane. The measurements were performed according to the protocol described elsewhere [36].

#### 2.4.10. Confocal Fluorescence Microscopy

Fluorescence microscopy images of UHMWPE films were collected using an Olympus IX81 fluorescence microscope (Olympus Corporation, Shinjuku City, Japan) with UV excitation and a Hamamatsu digital CCD camera C7780.

#### 2.4.11. Water Vapor Transmission Rate (WVTR) Measurements

Vapor permeability or water vaportransmission rate (WVTR) of the UHMWPE films was studied according to the Standard Test Methods ASTM E96 [37]. A cup was half-way filled with water. The sample was placed over the top of the cup and sealed with wax by contour, thus allowing the water vapor evaporation through the membrane. The cup was placed in a weather chamber (relative humidity 30%), and the total mass of the cup was periodically measured. WVTR reads as: *K =* Δ*m*/(*S* × *t*), or normalized by the film thickness *K =* Δ*md*/(*S* × *t*), where Δ*m* is the weight of the evaporated water vapor at time *t* (days), *S* is the membrane surface area, and *d* is the film thickness. The measurements were performed for, at least, three-five samples; the experimental error was 3–5%.

#### 2.4.12. Estimation of the Gurley Value

The Gurley value is known to be the basic property of porous materials which quantifies the air permeability of porous membranes as the time period required for the permeability of a given air volume (here, 100 cm^3^) through a membrane with an area of 1 in.^2^ at a given pressure (here, 0.02 MPa). Air permeability of the UHMWPE porous samples was measured according to the standard protocol [38].

## 3. Results

To justify the procedure allowing preparation of open-porous UHMWPE films with nanoscale pore dimensions via environmental crazing and to highlight the applied benefits of the advanced porous materials, this work involves the following steps: characterization of the structure/morphology and stress-strain behavior of the initial UHMWPE samples, the study of the mechanism of environmental crazing of the initial UHMWPE samples, quantitative description of the structural evolution of the UHMWPE films in the course of their tensile drawing via environmental crazing as the route for the design of porous organic materials, interpretation of the experimental results, and formulation of the optimal scenario providing the preparation of the high-performance porous materials with high shape stability. Preparation of porous organic materials with controlled morphology and desired properties requires a thorough characterization of the initial structure of the starting polymer, description of the criteria providing the development of environmental intercrystallite crazing, characterization of the resultant porous polymers as membranes, vapor and gas permeable breathable materials, gas storage materials, etc.

### 3.1. Preparation of Mesoporous Membrane Materials Based on UHMWPE Films via Environmental Crazing

#### 3.1.1. Structure and Morphology of Initial UHMWPE Films

**Figure 1:**
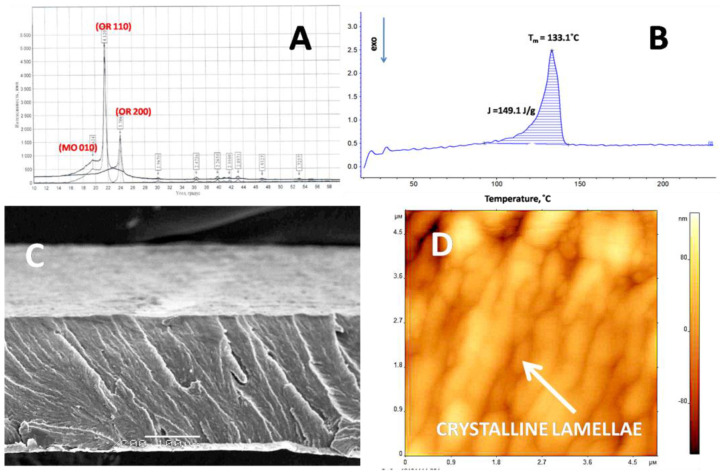
Structure and phase state of the initial UHMWPE films: (**A**) WAXS diffractogram, (**B**) DSC scan, (**C**) SEM image, and (**D**) AFM image.

Figure 1 presents the information on basic properties of the initial UHMWPE films. The initial structure of the UHMWPE film (Figure 1A) is characterized by the WAXS experiments. The WAXS diffractogram clearly shows the peaks attributed to the crystallographic planes (110) and (200), thus indicating the existence of a classical orthorhombic crystalline structure of polyethylenes. In addition to the X-ray reflections 110 and 200 corresponding to the orthorhombic crystalline lattice of UHMWPE (OR 110 and OR 200), the WAXS diffractogram shows a slight peak at θ = 9.6°, which is assigned to the monoclinic modification of UHMWPE (MO 010).

According to the DSC measurements (Figure 1B), the melting temperature of the UHMWPE films is 133.1 °C. The degree of crystallinity of the initial UHMWPE is calculated with respect to the heat of fusion of an ideal UHMWPE crystal (289 J/g [25]). Hence, the degree of crystallinity of the test films is equal to 51% (hence, the content of the amorphous phase is 49%). Glass transition temperature of the amorphous phase is −150 °C which means that, at the ambient temperature, the amorphous phase exists in the rubbery state (or highly elastic state). This fact means the structure of the sample can be presented as a composite two-phase system, which involves a rigid crystalline phase and a soft amorphous phase.

As follows from the corresponding SEM image (Figure 1C), the initial UHMWPE films are seen to be monolithic and contain no well-defined structural elements. This observation can be biased due to the low resolution of this method. Figure 1D shows the AFM image of the initial UHMWPE films. The structure of the semicrystalline UHMWPE is seen to be composed of the stacks of crystalline lamellae, and their average thickness is estimated to be equal to 30 ± 5 nm. Intercrystalline amorphous regions contain disordered polymer chains (load-bearing tie chains bridging neighboring crystalline lamellae, cilia, tails, loose loops, etc.) in the rubbery state.

To gain a deeper insight into the mechanism behind the effect of PALE on the UHMWPE, the degree of swelling of the polymer in organic solvents was studied. The test films were allowed to stay in the selected organic solvents (*n*-heptane, *n*-decane) until the equilibrium degree of swelling was attained. In the case of semicrystalline polymers, swelling can be treated as a certain mode of blending upon the penetration of an organic solvent into a polymer via diffusion. In the case of semicrystalline polymers, the diffusion of an organic solvent is provided exclusively by their penetration into amorphous regions whereas crystalline lamellae remain “non-transparent.” Hence, the organic molecules occupy only interlamellar amorphous phase. As a result of plasticization, glass transition temperature of the polymer decreases, and the amorphous regions become softer and more compliant. Evidently, the rate of diffusion and the degree of swelling depend on the properties of both solvent and polymeras well as on their interaction. As the content of the organic solvent within the interlamellar amorphous regions increases, the size of the above swollen regions gradually increases [39]. For aliphatic alcohols (*n*-butanol, IPA), the degree of swelling of the UHMWPE films is negligible. However, when hydrocarbons are used, the kinetics of swelling of UHMWPE is the following: within the first 1–15 min, the degree of swelling rapidly increases and then levels off, and the equilibrium degree of swelling is achieved within 25 min. For *n*-heptane, the equilibrium degree of swelling is 4.5 wt. % or 6.0 vol. %; for *n*-decane, 6.0 wt. % and 7.5 vol. %. The equilibrium degree of swelling reflects the content (or cargo) of the organic solvent in the polymer. Taking into account the fact that, for semicrystalline polymers, the low-molecular-mass solvent is accommodated within the amorphous phase, whereas the crystalline regions remains intact, it seems reasonable to re-calculate the equilibrium degree of swelling per the content of amorphous phase (in the case under study, 49% of the polymer volume). The equilibrium degree of swelling in the amorphous regions reads as:

(7) $$\alpha_{V}^{am}=\frac{\alpha_{V/}\rho_{1}}{\alpha_{V/}\rho_{1}+\left(1-\alpha_{V}\right)\left(1-æ\right)/\rho_{p}}$$

where $\alpha_{V}$ is the volume degree of swelling, æ is the degree of crystallinity of the polymer (according to the DSC scans), *ρ_p_* is the density of UHMWPE (0.853 g/cm^3^), and *ρ_l_* is the density of the PALE (*n*-heptane, 0.684 g/cm^3^ and *n*-decane, 0.730 g/cm^3^).

The normalized equilibrium degree of swelling of the amorphous phase of UHMWPE is equal to 16.5 and 19.0 vol. % for *n*-heptane and *n*-decane, respectively. Otherwise speaking, the amorphous phase is heavily loaded with by the low-molecular-mass organic liquid.

Therefore, the mechanical response of the semicrystalline UHMWPE films in the presence of the PALE should be controlled by the degree of crystallinity of the polymer, by the morphology of crystalline and amorphous regions and their combined response to the applied stress as well as by the interaction between the polymer and the environment.

#### 3.1.2. Mechanical Properties of Initial UHMWPE Films

**Figure 2:**
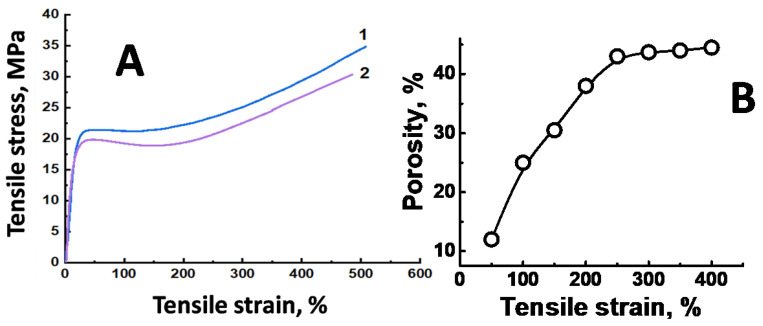
(**A**) Stress-strain curves illustrating the tensile drawing of the UHMWPE films in air (1) and in *n*-heptane and in the oil-in-water emulsions of *n*-heptane and water with a high water content of 95% (2) and (**B**) the plot describing the development of porosity of the UHMWPE samples with tensile strain upon tensile drawing in *n*-heptane and in the oil-in-water emulsions of *n*-heptane and water with a high water content (95% of water).

Let us consider in more detail the behavior of the UHMWPE films upon tensile drawing. Figure 2 shows the corresponding stress-strain curves of the UHMWPE films upon their tensile drawing in air and in the PALE.

First, let us discuss the visual appearance of the samples in the course of their tensile drawing in air and in the PALE. Deformation of the UHMWPE films in air is uniform with a visual side contraction and a marked loss in the film thickness. Deformation of the UHMWPE films in the presence of the PALE (*n*-heptane) proceeds at much lower stress level and without any side contraction. As compared with deformation in air (Figure 2A, curve 1), elastic modulus of the UHMWPE films markedly decreases (from 530 to 300 MPa), and this fact can be reasonably explained by the plasticization of the amorphous regions. Yield stress and post-yield stress are also seen to be lower. This fact well agrees with the general pattern of deformation of other polyolefins [32,34,40]. As follows from Figure 2A, deformation of the UHMWPE films proceeds to high tensile strains *ε* up to the stage of orientational strengthening.

Let us mention that the O/W emulsions with high water content (95 vol. %) can serve as efficient PALE for UHMWPE: as follows from Figure 2, the corresponding stress-strain curve and porosity-(tensile strain) plot appear to be identical for the UHMWPE samples whether the films are stretched in *n*-heptane or in the corresponding O/W emulsion with high water content (95 vol. %). This observation proves the efficiency of the O/W emulsions with high water content as an ecologically safe alternative to organic PALE and the mechanism of the action of the O/W emulsions on polymers has been described in full detail [41]. Hereinafter, for ecological safety, only O/W emulsions with high water content were used as the PALE.

#### 3.1.3. Structural Evolution of Initial UHMWPE Films upon Environmental Crazing

**Figure 3:**
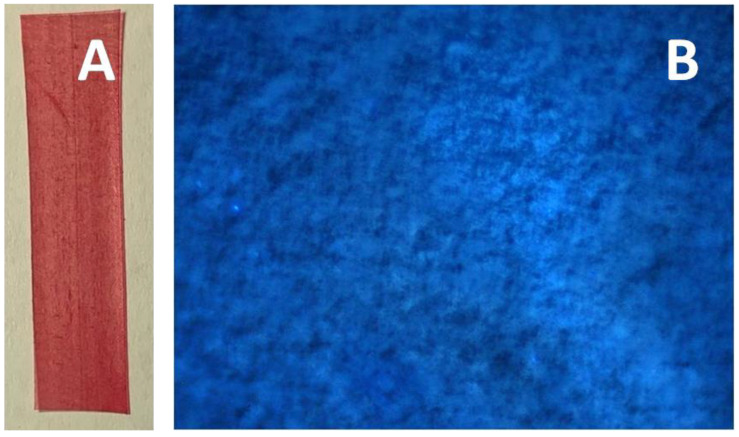
(**A**) Optical micrograph of the UHMWPE films after tensile drawing in O/W emulsion based on *n*-heptane and water (95 vol. %) contrasted by the oil-soluble dye Sudan IV (dye-contrasting tests) and (**B**) confocal fluorescence micrograph of the UHMWPE films after tensile drawing in *n*-heptane and dye-contrasting by the fluorescent dye Rhodamine 6G in IPA. Tensile strain is 250%.

Figure 2B shows that, upon tensile drawing in the PALE, porosity of the UHMWPE samples gradually increases and achieves 45% at a tensile strain of 250%. To reveal whether the porosity is uniform, the UHMWPE samples were stretched in the presence of the dye-containing solution (Sudan IV) when the oil-soluble dye was dissolved in *n*-heptane. Figure 3 presents the optical micrograph of the UHMWPE sample with *ε* = 250%.

As follows from Figure 3A, the UHMWPE samples are seen to be uniformly and brightly colored, and this fact suggests that the dye penetrates the as-formed porous structure in the course of the tensile drawing. When the sample after stretching is immersed in the dye-containing solution, the sample also becomes colored. To visualize the distribution of the dye within the whole volume of the sample (throughout the film thickness), the method of confocal fluorescence microscopy was used when the sample was scanned across the thickness (Figure 3B). The microscopic observations prove that the distribution of the dye is uniform throughout the whole volume.

**Figure 4:**
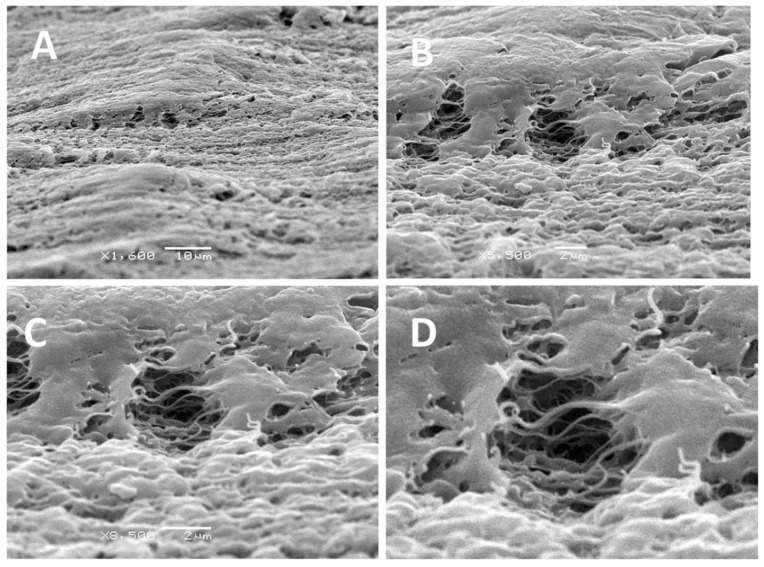
SEM images of the UHMWPE films after their stretching in the PALE (O/W emulsion based on *n*-heptane and water, 95 vol. % of water) by tensile strain *ε* = 250% ((**A**,**B**)—surface of the sample; (**C**,**D**)—enlarged images).

**Figure 5:**
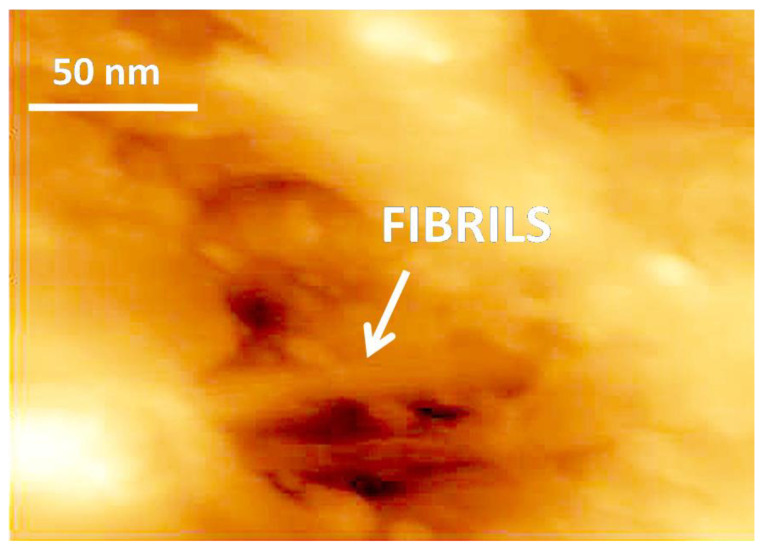
AFM image of the UHMWPE film after tensile drawing in the PALE (O/W emulsion based on *n*-heptane and water, 95 vol. % of water) by tensile strain *ε* = 250%.

Figure 4 and Figure 5 shows the SEM and AFM images of the UHMWPE samples after their tensile drawing in the PALE.

As follows from Figure 4 and Figure 5, the UHMWPE films after their tensile drawing in the PALE are seen to contain multiple pores. The pores are accommodated within the intercrystallite regions between crystalline lamellae. The specific feature of this structure is concerned with the fact that the amorphous phase is organized as asymmetric fibrils oriented along the stretching direction, and the fibrils with a thickness of 10 nm are spaced by pores with nanoscale dimensions. The fibrils bridge the neighboring crystalline lamellae which preserve their thickness and remain intact upon tensile drawing up to 300% and, seemingly, can serve as reinforcing elements for the structure in whole.

This evidence unequivocally suggests that deformation of the UHMWPE films in the presence of the PALE proceeds via the mechanism of environmental intercrystallite crazing [32,33,34,40]. Let us mention that deformation of the UHMWPE films in air does not assist the development of any porosity, and the crucial role in this process belongs to the PALE. The role of the PALE is multipurpose (at least, triple) as the PALE provides the plasticization of amorphous regions and makes them softer and more compliant. As a result, the highly elastic softened phase is confined between rigid bodies (crystalline lamellae). A huge difference between the mechanical properties of rigid crystalline lamellae and softened amorphous phase provides the necessary prerequisite for triggering the Raleigh-Taylor meniscus instability phenomenon [42] and stress-induced fibrillation (fingering) when the rigid plates with a confined soft phase (a classical Hele-Shaw cell [43]) are driven apart. Upon tensile drawing of the polymer films, PALE also serves as a lubricant, thus assisting efficient separation of crystalline lamellae as they are drifted apart [39]. Finally, efficient separation of crystalline lamellae is accompanied by fibrillation and cavitation and, when the highly porous structure is formed, the PALE adopts a charitable role of a surface-active agent (a surfactant).

#### 3.1.4. Structural Parameters of Mesoporous UHMWPE Materials

Structural parameters of the as-drawn UHMWPE films were studied by the efficient method of pressure-driven liquid permeability (PDLP). Let us mention that the as-drawn “native” UHMWPE films are permeable to IPA, and this fact lends the additional evidence that the pathways providing the mass transport along the membrane are free, and the as-formed porous structure is open. The IPA flow of the UHMWPE films increases by a factor of eight as the tensile strain increases from 100% to 250% from 0.05 L/(m^2^ × h) (*ε* = 100%) to 0.38 L/(m^2^ × h) (*ε* = 250%).This fact suggests that the porous UHMWPE materials can be used as membranes for liquid permeability.

**Figure 6:**
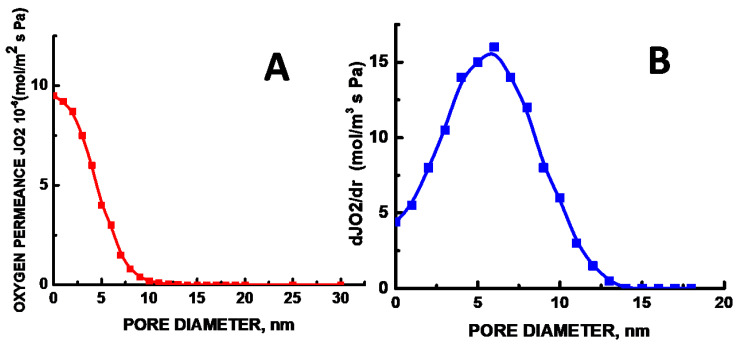
(**A**) Oxygen permeance and (**B**) pore size distribution of the UHMWPE materials with tensile strain *ε* = 250%.

Knowing the IPA flow and porosity, average pore diameter *D*_p_ can be calculated using Equation (6). The average pore diameter *D*_p_ of the stretched UHMWPE samples increases with increasing the tensile strain from 2 nm (*ε* = 100%) to 5.5 nm (*ε* = 250%). According to Equation (5), the estimated number of pores per unit area is equal to n = 7 × 10^17^ m^−2^. This estimation is also supported by the results of permporometry (Figure 6).

Hence, according to International Union of Pure and Applied Chemistry (IUPAC), the prepared UHMWPE films can be classified as mesoporous (MP) materials [12]. As follows from visual microscopic observations (Figure 4 and Figure 5), all pores are seen to be interconnected. The proposed approach of environmental crazing can be envisaged as a facile and reliable route for the design of MP UHMWPE materials with controlled and uniform porosity, interconnected pores, high surface area, and nanoscale pore dimensions (below 10 nm). Noteworthy is that this process proceeds at room temperature and can be controlled by varying the tensile strain *ε* and conditions of tensile drawing, thus allowing preparation of MP materials with desired porosity and pore diameter.

#### 3.1.5. Preparation of Mesoporous Materials with High Shape Stability

**Figure 7:**
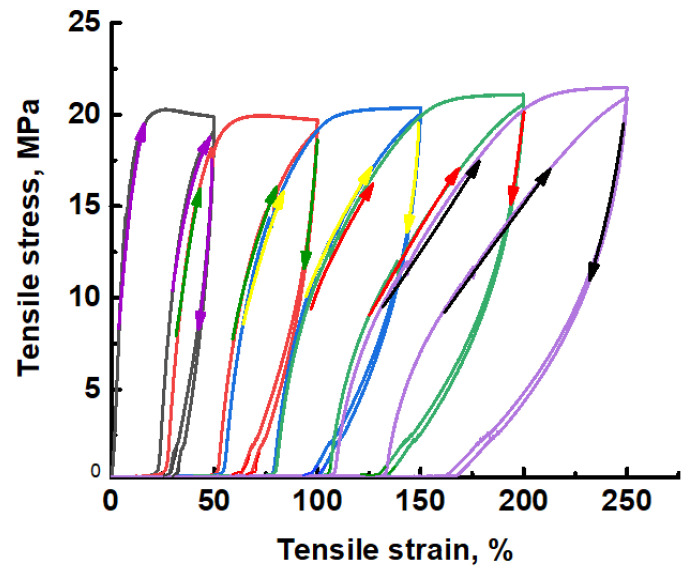
Stress-strain curves illustrating the cyclic loading/unloading for the UHMWPE films in the PALE (O/W emulsion based on *n*-heptane and water, 95 vol. % of water).

The proposed approach allows preparation of MP UHMWPE materials with high porosity and nanoscale pore dimensions. However, immediately after environmental crazing, these materials are characterized by low shape stability. Figure 7 shows the stress-strain curves illustrating the cyclic loading/unloading of the UHMWPE films in the presence of the PALE.

As follows from Figure 7, when the sample is stretched by a given tensile strain and then unloaded in a free state, the length of the sample along the stretching direction is reduced, and the sample shrinks down. The sample exhibits this strain recovery even being unloaded from high tensile strains (*ε* = 250%). This process is referred to as the low-temperature strain recovery (LTSR), and this behavior is typical of so-called hard elastic materials [44].

Equilibrium strain recovery of the stretched UHMWPE samples is estimated as follows: the samples after stretching are allowed to stay in a free-standing state, and their length is monitored. Equilibrium strain recovery is achieved within 40 min even though the sample quickly shrinks down within the first 5–7 min (80%) and the remaining 20% take another 30 min. For the UHMWPE sample with *ε* = 100%, strain recovery is ~87%; in other words, the sample recover its initial dimensions.

This behavior can be explained as follows. All solids with high porosity and nanoscale pore dimensions are characterized by an excessive surface energy and tend to reduce the energy via diverse scenarios. For the MP UHMWPE samples under study, the driving force behind the LTSR process is concerned with the ability of the system to reduce the surface energy via folding of nanoscale fibrils and shrinkage [34]. Hence, the amorphous phase as asymmetric fibrils is responsible for high shape instability and can ensure the marked decrease in excessive surface energy.

Hence, to prevent this undesirable phenomenon of LTSR and to prepare the mesoporous UHMWPE materials with high shape stability, the procedure of thermal stabilization is applied. To this end, the stretched samples after their tensile drawing in the PALE were dried to remove the PALE by evaporation and subjected to annealing at a temperature below the melting temperature of UHMWPE. The necessary condition is that this procedure should be performed under isometric conditions when the dimensions of the samples are fixed either by contour or along the direction of extension. The annealing temperature was selected according to the thermomechanical tests: the optimal annealing temperature appears to be 120 °C. After annealing, the samples become stable and fully preserve their dimensions (gage size as length, width, and thickness); porosity and pore size remain unchanged (in comparison with “native” samples immediately after stretching). Let us mention that the degree of crystallinity of the annealed UHMWPE samples is similar to that of the initial UHMWPE. The tests on the shape stability of the annealed samples were performed in a broad temperature interval (below the melting temperature of UHMWPE) when the ink dots were deposited onto the surface of the test sample and the distance between them was measured after the free-standing samples were heated to a given temperature. The experiments show that, at temperatures below 90 °C, all samples fully preserve their dimensions and show high shape stability. Let us mention that, according to the DSC tests, this point corresponds to the onset of melting. As is expected, at higher temperatures, the shrinkage of the samples increases and, finally, at the melting temperature, the sample melts down. This phenomenon of the acquired shape stability can be explained as follows: upon the tensile drawing of the UHMWPE films in the PALE via intercrystallite environmental crazing, the neighboring crystallites remain intact but interconnection between crystallites is lost, and the whole crystalline framework appears to be disconnected (thus leading to the shrinkage when the stretched samples are set free); upon annealing at temperatures below the melting temperature, interconnection between disconnected crystallites is restored due to the partial recrystallization and formation of so-called tie-crystals, which bridge the neighboring lamellae; the as-formed interconnected network of crystallites serves as a strong skeleton which makes it possible to preserve the porosity of the polymer samples, and the resultant materials are characterized by high shape stability.

Finally, in addition to high porosity and shape stability, porous materials should be mechanically strong and robust. When comparing the mechanical characteristics of the initial UHMWPE films and MP UHMWPE samples (porosity is 45%), the elastic modulus decreases only by 15% (from 530 down to 480 MPa), and the yield point decreases from 21.4 MPa down to 17.6 MPa. This fact can be explained by the high rigidity of intact crystallites as well as by high orientation of macromolecular chains within the fibrils bridging the neighboring crystallites.

To conclude, tensile drawing of the semicrystalline UHMWPE samples in the presence of the PALEs is accompanied by the development of a marked macroscopic porosity via intercrystallite environmental crazing, and the average pore diameter of the samples lies within the nanoscale interval below 10 nm. This approach offers a facile and reliable strategy for the preparation of highly porous UHMWPE materials with nanoscale pore dimensions and interconnected pores, and these materials may be useful in diverse practical applications.

### 3.2. Applied Properties of Mesoporous UHMWPE Membrane Materials

#### 3.2.1. Air Permeability of Mesoporous UHMWPE Materials

For all porous materials, air permeability defined as the Gurley value is known to be one of the basic parameters, especially when the porous materials are used as biomedical materials and microporous separators in primary (single-use) and secondary (rechargeable) lithium batteries (LIB) [8,45,46,47]. The Gurley value is the air permeability expressed as the time required for a given volume of air (100 mL) to pass through a given area of the separator under a given pressure [8]. For the MP UHMWPE films stretched by 250%, the Gurley value is 300 s, and this value appears to be in a good agreement with the reported data for the commercial HDPE and PP membrane separators (including Celgard separators) [47].

#### 3.2.2. Vapor Permeability of Mesoporous UHMWPE Materials

Vapor permeability or water vapor transmission rate (WVTR) of the membrane materials is the basic characteristic of the porous polymeric materials and serves as a guideline demonstrating the applied value of the proposed membranes. All WVTR tests were performed according to the standard procedure and for the MP samples with the maximum porosity (*ε* = 250%). Under the testing conditions, WVTR of the MP UHMWPE samples with *ε* = 250% (film thickness is 130 microns) was 1700 g/(m^2^ × day). This value (being normalized by film thickness) appears to be comparable to that of the commercial porous membranes.

The resultant MP UHMWPE materials are also characterized by high water entry pressure. The water entry pressure was estimated according to the Young-Laplace equation [48]: *P* = (2γ × cosθ)/r, where *P* is the water entry pressure, *γ* is the surface tension of water (0.07197 N m^−1^ at 25 °C), *r* is the pore radius (according to the PDLP tests), *θ* is the wetting contact angle (140° for the MP UHMWPE). As a result, the water entry pressure of the MP UHMWPE is ~250 bar. In other words, the resultant materials are water-proof as the water entry pressure is very high but show high water vapor permeability (breathable materials).

To gain a deeper insight into the potential of the MP UHMWPE films as selective membrane materials, vapor permeability of ethanol was studied. Ethanol vapor transmission rate is measured to be equal to 6300 g/(m^2^ × day); in other words, this value is 3.7 times higher than that of water. Then, selectivity of the MP UHMWPE membrane can be roughly estimated as *S = Q_EtOH_*/*Q_w_* = 3.7 where *Q_w_* and *Q_EtOH_* stand for vapor transmission rate of water and ethanol, respectively.

#### 3.2.3. Effective Thermal Conductivity

In mesoporous polymeric materials, a gaseous phase is entrapped within nanoscale pores, and thermal conductivity of the gaseous phase is markedly depressed in full accordance with the well-known Knudsen effect [4] when pore scale is comparable or smaller than the mean free path of the gas. The effective thermal conductivity *λ*′*_g_* of the gas in air-filled porous solids is described by the Knudsen equation:

(8) $$\lambda^{\prime }g=\frac{{\lambda^{\prime }}_{g0}}{1+\beta \left(\frac{l_{g}}{D}\right)}$$

where *λ*′*_g_*_0_ is the thermal conductivity of free air (0.026 W/m K at room temperature), *β* is a parameter that takes into account the energy transfer between gas molecules and the limiting solid structure (about 2 for air), *l_g_* is the mean free path of the gas molecules (*l_g_* = 70 nm at room temperature), and *D_p_* is the average pore diameter. According to the calculations, the effective thermal conductivity *λ*′*_g_* of air in the MP UHMWPE materials is equal to 0.00107 W/(m K) and this value is nearly ~30 times lower than that of free air. Let us mention that this equation neglects the tortuosity of the mesoporous UHMWPE materials whereas, as follows from Figure 6, the MP UHMWPE materials are seen to be highly tortuous. High tortuosity together with nanoscale dimensions of pores and fibrils provides high specific surface of the samples, which is vitally important for their use as filters, membranes, insulating materials, materials for sound absorption, etc. Evidently, thermal insulation of the solids with well-pronounced tortuosity is enhanced.

## 4. Discussion

Preparation and targeted design of porous organic materials with predictable morphology and controlled pore dimensions from the scratch demands scientific description and justification of all involved stages, starting from the characterization of initial materials and the mechanism of their structural modification up to the culminating stage related to the elucidation of their practical benefits. In this work, the potential of environmental crazing was studied as an avenue towards the design of the UHMWPE-based porous materials. Taking into account the fact that UHMWPE is an attractive candidate from the viewpoint of membrane science and technology and the techniques allowing the processing of UHMWPE into porous materials are limited, this work highlights the advantages of environmental crazing for the stress-induced development of nanoscale porosity.

From the scientific viewpoint, UHMWPE satisfies all prerequisite conditions providing the development of porosity via environmental crazing as UHMWPE is a typical semicrystalline polymer from the family of polyethylenes with a high degree of crystallinity. Coexistence of a rigid lamellar structure of the crystalline phase and a softened amorphous phase in a rubbery within the intercrystallite regions triggers the stress-induced onset and progress of the Raleigh-Taylor meniscus instability phenomenon (cavitation and fibrillation). Moreover, this process is enhanced by swelling and plasticization of the amorphous phase by organic solvents. As a result, overall porosity of the UHMWPE films can achieve 45% and can be controlled by the tensile strain (Figure 2).

Pore size depends on the size of crystalline lamellae; this parameter increases with tensile strain and lie within the scale interval below 10 nm. In this study, the development of a nanostructured fibrillar morphology and a system of interconnected open pores via environmental crazing has been proved by diverse physicochemical methods, including SEM, AFM, dye-contrasting tests, liquid permeability, and permporometry.

To mitigate the environmental crazing of UHMWPE in the presence of toxic organic solvents and to advance an ecologically safe protocol, a “green” approach was applied when organic solvents (in the case under study, *n*-heptane) are substituted by the corresponding oil-in-water emulsions with high water content (above 95%). With respect to UHMWPE, the O/W emulsions with microscale oil droplets behave as pure solvents, and the development of porosity via environmental crazing is fully preserved.

The as-formed porous structure of UHMWPE with nanoscale pore dimensions is characterized by high shape instability and free-standing samples shrink down and nearly resume their initial dimensions (low-temperature strain recovery). Taking into account the driving force of this shrinkage as the tendency to reduce the excessive surface energy, this structure can be stabilized by the removal of the organic solvents from the amorphous phase (by evaporation) and reconstruction of the crystalline interconnected framework between disconnected crystalline lamellae via annealing of the samples under isometric conditions.

**Figure 8:**
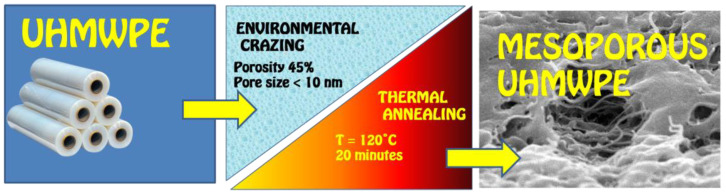
Scheme illustrating the strategy of environmental crazing for the preparation of the mesoporous UHMWPE membrane materials.

Hence, the mechanism of environmental crazing of UHMWPE films can serve as a scientific platform for the preparation of mesoporous UHMWPE materials with high shape stability and good mechanical properties (Figure 7 and Figure 8).

As a result of environmental crazing and annealing, mesoporous materials based on UHMWPE with high shape stability can be prepared, and their applied benefits are highlighted.

## 5. Conclusions

**Figure 9:**
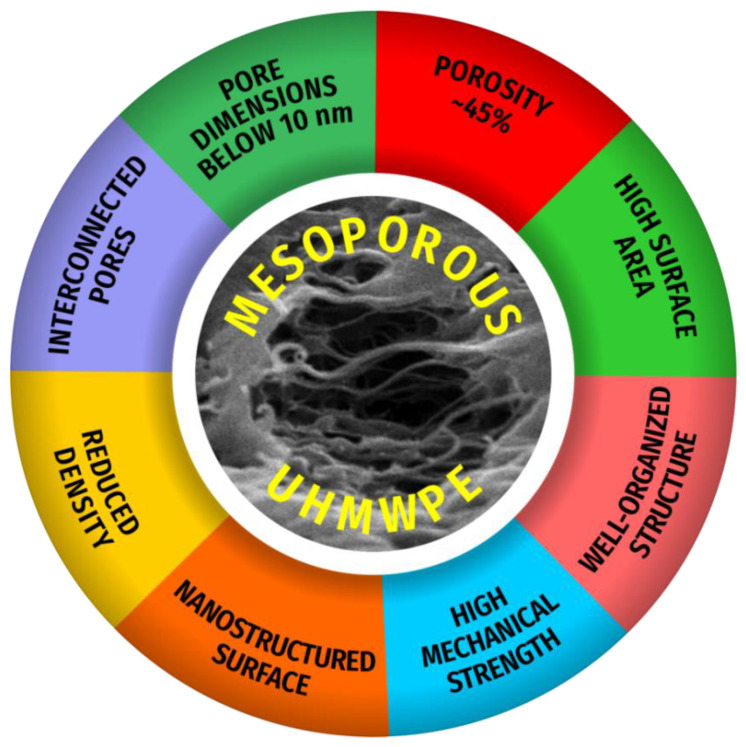
Structural characteristics of MP UHMWPE materials.

To conclude, tensile drawing of the semicrystalline UHMWPE samples in the presence of the PALEs proceeds via the mechanism of environmental crazing and is accompanied by the development of a marked macroscopic porosity, and the average pore diameter of the samples lies within the nanoscale window (below 10 nm). The proposed approach allows preparation of mesoporous UHMWPE materials with an open uniform porosity, interconnected pores, high surface area, nanoscale pore dimensions, high shape stability, and good mechanical properties (Figure 8 and Figure 9).

Evident technological profit of the proposed approach is related to the fact that this process can be performed at room temperature (cold process) and can be upscaled using the conventional technological equipment for the orientational drawing of polymers. Moreover, this process can be used for films, tapes, fibers, nonwovens, etc. Our further studies in this direction will be devoted to this challenge (especially, when passing from films to fibers as the samples with an alternative geometry).

**Figure 10:**
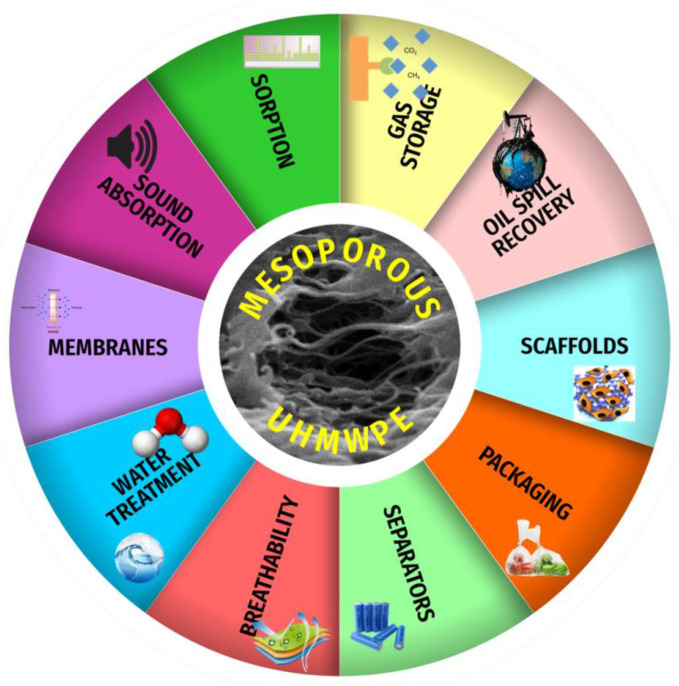
Potential areas of practical application of the MP UHMWPE materials.

From the practical viewpoint, the resultant MP UHMWPE materials seem to be promising candidates for their potential use as applied materials with valuable functional properties: they are characterized by high air permeability (low Gurley value), high water vapor transmission rate, breathability coupled to waterproof characteristics, good insulating properties, etc. The MP UHMWPE materials can be used as mesoporous membranes, gas storage systems, vapor permeable materials, light-weight materials, “breathable” materials, battery separators [8], filters, scaffolds for tissue and cell engineering (nanoscale level for protein conformation) [45], nanoporous substrates [45], sorbents, precursors for porous carbons [7], sound absorption materials [49], sorption and oil spill recovery [19,20,50], gas capture and storage [5], materials for biomedical applications, etc.(Figure 10).

Of special interest is the application of MP UHMWPE materials as porous substrates and matrixes for the preparation of nanocomposite materials via introduction of various functional additives into the inner nanoscale cavities. Depending on the nature of the additive, the functionalized materials can be used for heterogeneous catalysis with high regioselectivity and stereoselectivity as well as antibacterial materials, sensors, materials with controlled hydrophilic/hydrophobic balance, etc.

## Data Availability

Data used in this article were obtained from the scientific literature; references given below.
